# Synthetic dataset generation of energy consumption for residential apartment building in cold weather considering the building's aging

**DOI:** 10.1016/j.dib.2024.110445

**Published:** 2024-04-20

**Authors:** Juan Pablo Diaz Ramirez, Shaival Hemant Nagarsheth, Camilo Enrique Ducuara Ramirez, Nilson Henao, Kodjo Agbossou

**Affiliations:** Laboratoire d'innovation et de recherche en énergie intelligent (LIREI), Institut de recherche sur l'hydrogène (IRH), Université du Québec à Trois-Rivières (UQTR), Québec G9A 5H7, Canada

**Keywords:** Energy consumption dataset, Energy demand, Electric baseboard heater, Multi-unit residential building, Winter data, Indoor climate

## Abstract

The residential sectorʼs substantial electricity consumption, driven by heating demands during winter, necessitates optimal energy consumption strategies in the era of decarbonization. To address this challenge, this paper introduces a synthetic dataset specifically tailored to simulate energy consumption in residential apartment buildings. Focusing on the interplay of cold weather conditions and the effects of aging factors, the dataset comprehensively encompasses key variables, including indoor temperature, energy consumption, outdoor temperature, outdoor humidity and solar radiation. It underscores the considerable impact of building aging on energy consumption patterns. The datasetʼs significance extends across various domains, particularly in the realms of energy forecasting and thermal modelling. It serves as a robust foundation for predicting future consumption patterns, optimizing resource allocation, and refining energy efficiency strategies. The inclusion of indoor temperature data facilitates an in-depth thermal modelling approach, shedding light on intricate relationships that influence building performance in cold climates. Beyond traditional, the dataset proves invaluable in nonlinear modelling and machine learning. It emerges as a key tool for algorithm training, enhancing forecast precision, and supporting well-informed decision-making. The introduction of a temporal dimension by accounting for aging factors allows for the exploration of evolving building components over time, a critical consideration for sustainable energy management and building maintenance strategies. The dataset was meticulously generated by creating geometry using SketchUp and conducting energy modelling and simulations via the OpenStudio platform, which integrates the Energy Plus modelling engine to enhance accuracy. In summary, this synthetic dataset generation provides valuable insights into energy consumption in residential buildings exposed to cold weather conditions and the influences of aging. Its multifaceted applications across forecasting, modelling, management, and planning underscore its potential to advance sustainable and efficient energy practices.

Specifications Table

**Table:** 

Subject	Energy
Specific subject area	Energy Engineering and Power Technology
Data format	Raw
Type of data	Synthetic dataset stored in .csv files
Data collection	The data collection process involved a three-tiered approach. Geometry creation utilized SketchUp, ensuring regional alignment. OpenStudio for whole-building energy modelling using EnergyPlus for construction materials, building specifications and data sampling granularity. Rigorous adherence to ASHRAE standards and cross-referencing ensured data accuracy. This holistic approach provides a reliable dataset for electricity power demand analysis.
Data source location	Université du Québec à Trois-Rivières, Trois-Rivières, Québec, Canada Geographical Coordinates - 46.3470° N, 72.5785° W
Data accessibility	Repository name: Harvard Dataverse Data identification number: 10.7910/DVN/SPNY8U Direct URL to data: https://doi.org/10.7910/DVN/SPNY8U Instructions for accessing these data: None

## 1 Value of the Data

• The dataset generated for a residential building in a cold climate encompasses variables such as indoor temperature, humidity, energy consumption, outdoor temperature, solar radiation, outdoor humidity, and aging factors. This dataset holds immense value for energy system researchers in the fields of energy forecasting and thermal modelling. These researchers can leverage this data to predict and gain insights into energy consumption patterns. Their work enables stakeholders to optimize resource allocation and enhance energy efficiency, contributing significantly to the development of sustainable energy solutions.
• This dataset can be useful for energy modellers, policymakers, utility companies and other related stakeholders for achieving a comprehensive modelling approach, facilitating a nuanced understanding of how these factors influence energy usage and building performance in cold climates.
• For machine learning researchers, as the data is open-source data, it helps overcome challenges in data generation for forecast modelling. Moreover, this data is crucial for training algorithms to predict energy demands, enhancing forecast accuracy and aiding informed decision-making for energy analysts.
• Accounting for aging factors in the dataset adds a time dimension, enabling policymakers and practitioners to study the evolution of building components and their impact on energy consumption trends. This time-related aspect underscores the dataset's uniqueness and regional relevance, playing a crucial role for building managers in devising sustainable energy management strategies.
• In the broader context of energy management and demand response programs, the dataset becomes instrumental in devising proactive strategies to balance energy supply and demand. By leveraging the insights derived from the dataset, energy management organizations can implement targeted practices and participate in demand response programs more effectively, contributing to grid stability and overall energy sustainability.
• For professionals working on transactive energy platforms, this dataset becomes a valuable resource for understanding the temporal variability in energy demand, aiding in the development of innovative pricing mechanisms and market strategies. By incorporating this dataset into transactive energy models, stakeholders can optimize energy transactions, enhance grid reliability, and foster a more resilient and responsive energy ecosystem.
• The dataset [1] is instrumental for thermal and energy systems engineers in forecasting the dimensions and planning of thermal systems using energy demand data. By understanding the correlation between indoor environmental conditions and energy usage, engineers can make knowledgeable decisions about the design and capacity of HVAC systems. This ability is vital for optimizing thermal system performance, reducing energy wastage, and advocating for energy-efficient solutions customized to the unique needs of buildings in colder climates.

## 2 Background

This dataset generation motivation stems from the imperative transition of electric grids due to escalating electricity demand driven by the global need for decarbonisation. This shift aims to minimize environmental and societal costs associated with energy production, consumption, and management. Focusing on the residential sector, where heating constitutes 54 % of energy demand [2] in Quebec, Canada, it is crucial to perform a study on energy optimization [3]. The data generation related to indoor heating profiles through electric baseboard heaters is essential to identify and characterize the different heating needs. Comprehensively, it aids in understanding and analysing the dynamics of energy usage, facilitating the creation of machine learning-based models for the energy management infrastructure of buildings.

Moreover, understanding the thermal transmittance of the building envelope is vital for assessing heating demand and ensuring thermal comfort [4]. This property corresponds to the building's performance and insulation capacity, susceptible to deterioration over time [5]. Various factors contribute to this aging process, including air cavities, moisture content, material quality, and prolonged exposure to weather conditions [6]. Thus, the purpose of synthetically generating an energy consumption dataset by considering aging is to help create predictive models and long-term planning [7].

To overcome challenges in acquiring real-world data, synthetic data [7,8] and building modelling emerge as viable strategies for energy analysis, demand response scenarios and energy planning. Importantly, it is estimated that by 2030, synthetic data will overtake actual data in training AI models [8]. The intent is to provide a foundation for further analysis, offering a resource that complements original research articles by facilitating investigations into energy optimization and the impacts of residential apartment building envelope characteristics on energy consumption.

## 3 Data Description

To generate the dataset, a residential building decomposed into 10 thermal zones is considered. Two types of data are generated and provided in the present synthetic dataset: (i) Building data in winter and (ii) Aging data. Building data in the winter season with a 1-min granularity from the 1st of January to the 28th of February is generated, where various variables, namely, indoor temperature, energy consumption by electric baseboard heaters, and thermostat setpoint, are incorporated. Additionally, the weather data, including the outdoor temperature and solar radiation, is also embedded.

**Fig. 1:**
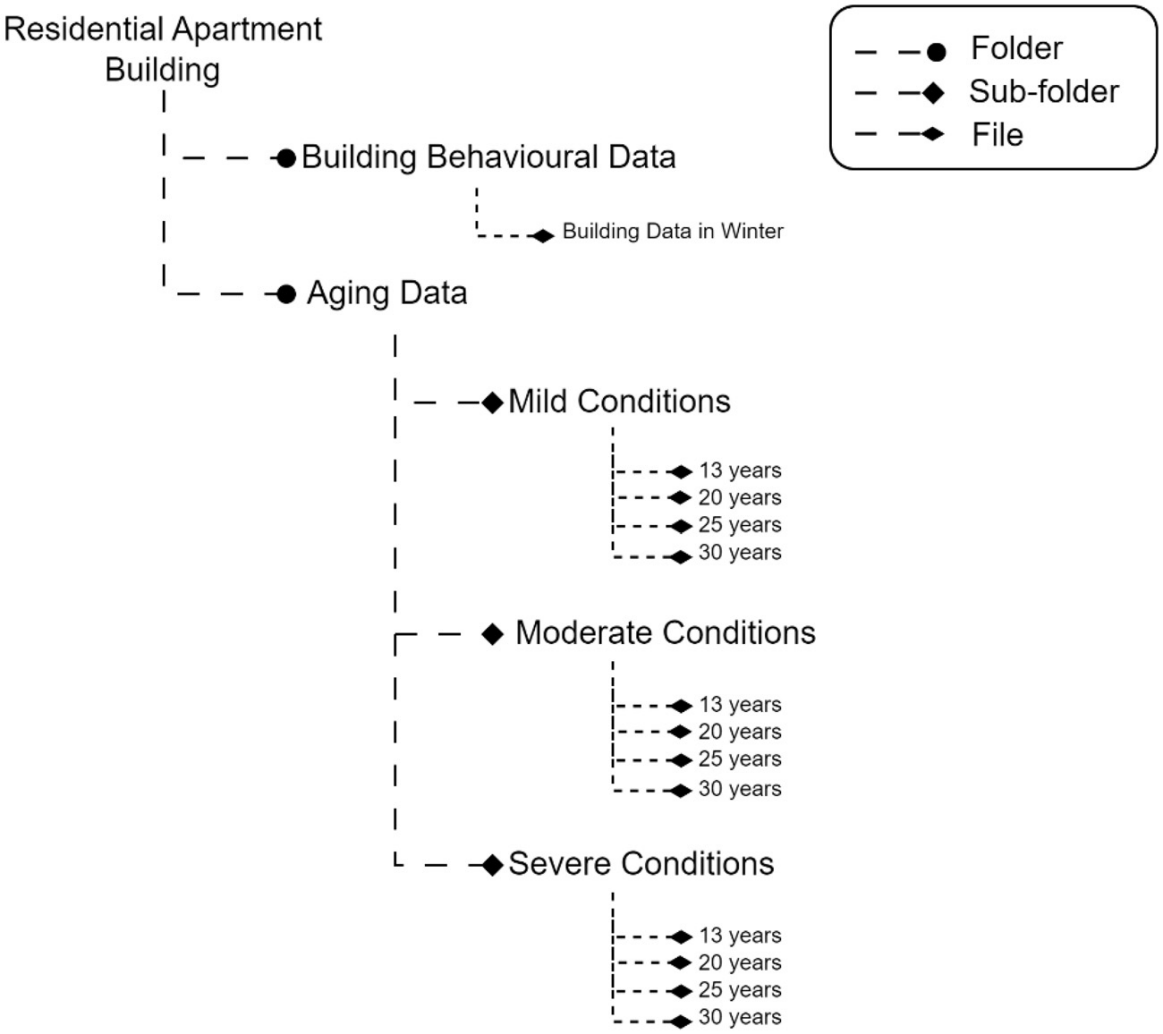
Dataset folder distribution in the repository.

For energy consumption data with an aging factor (aging from 13 years to 30 years), data is generated with an hourly granularity from the 1st of January to the 28th of February. Fig. 1 shows the bifurcation of the data repository with its folders, subfolders and files. Moreover, Table 1 shows the data columns with descriptions.

**Table 1:** An outline of the variables in the dataset.

Name of the columns (Variables)	Description
EBH Living Room Apt i	Baseboard Electricity Consumption (kWh) of the living rooms
EBH Chamber Apt i	Baseboard Electricity Consumption (kWh) of the chambers
EBH Corridor j	Baseboard Electricity Consumption (kWh) of corridors of the building
SP Living Room Apt i	Thermostat Setpoint (°C) of the living room of each apartment
SP Chamber Apt i	Thermostat Setpoint (°C) of the chamber of each apartment
SP Corridor j	Thermostat Setpoint (°C) of corridors of the building
Temp Living Room Apt i	Indoor Temperature (°C) of the living room of each apartment
Temp Chamber Apt i	Indoor Temperature (°C) of the chamber of each apartment
Temp Corridor j	Indoor Temperature (°C) of Corridors of the building
Outdoor Temp	Outdoor Temperature (°C) of Trois-Rivières, QC Canada
Outdoor Solar Radiation	Outdoor Solar Radiation (kW/m^2^) of Trois-Rivières, QC Canada

i=1to 10,and j=1,2

## 4 Design, Materials and Methods

**Fig. 2:**
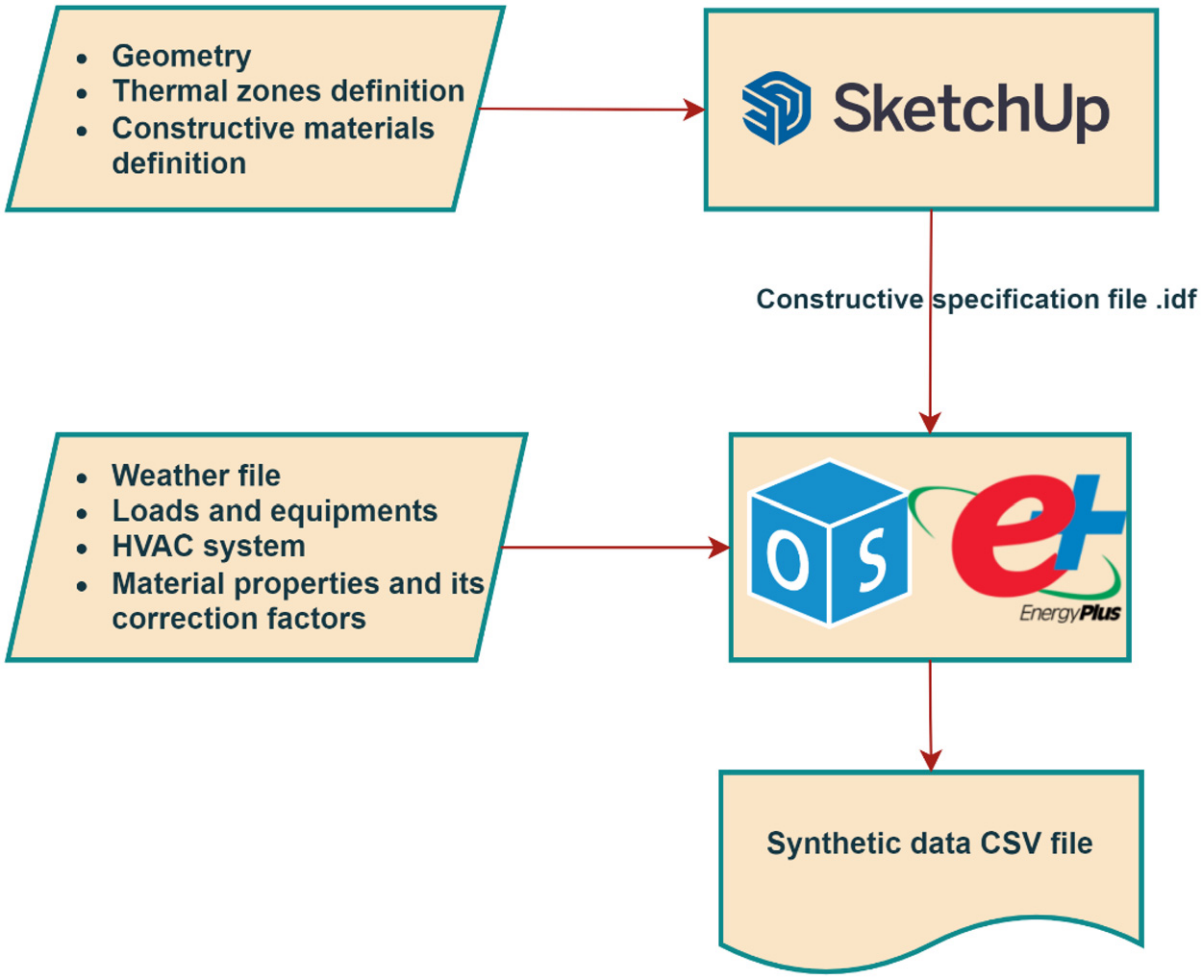
Numerical Flow to generate synthetic data.

To generate the data, open-source platforms have been utilized. Fig. 2 depicts the connections between the platforms used to design the structure for the simulation.

The graphical interfaces of SketchUp and OpenStudio [9] facilitate detailed modelling of the proposed work due to its compatibility with the EnergyPlus simulation Engine [10]. This design methodology facilitates achieving a high level of detail in the thermal dynamics of the buildings to obtain close-to real-life behaviour in terms of energy consumption and indoor environment.

**Fig. 3:**
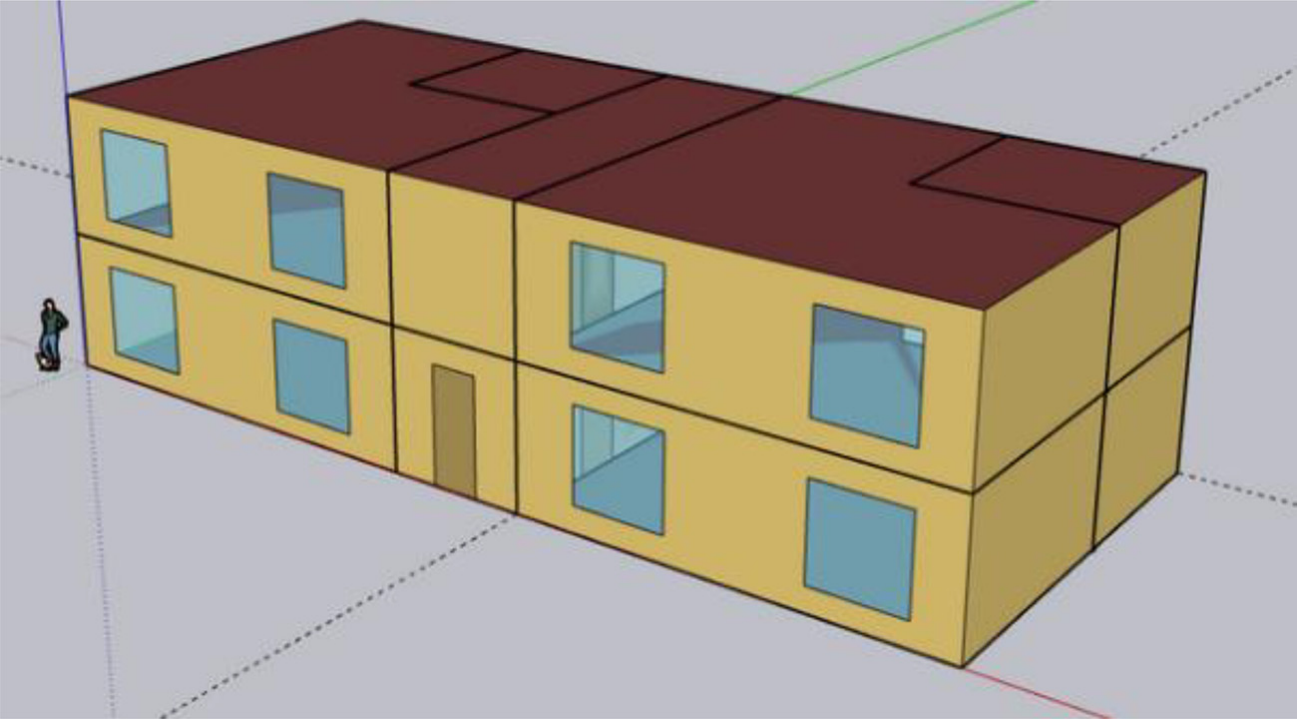
4 units– 10 zones residential apartment building in SketchUp for Trois-Rivieres, QC, Canada.

**Fig. 4:**
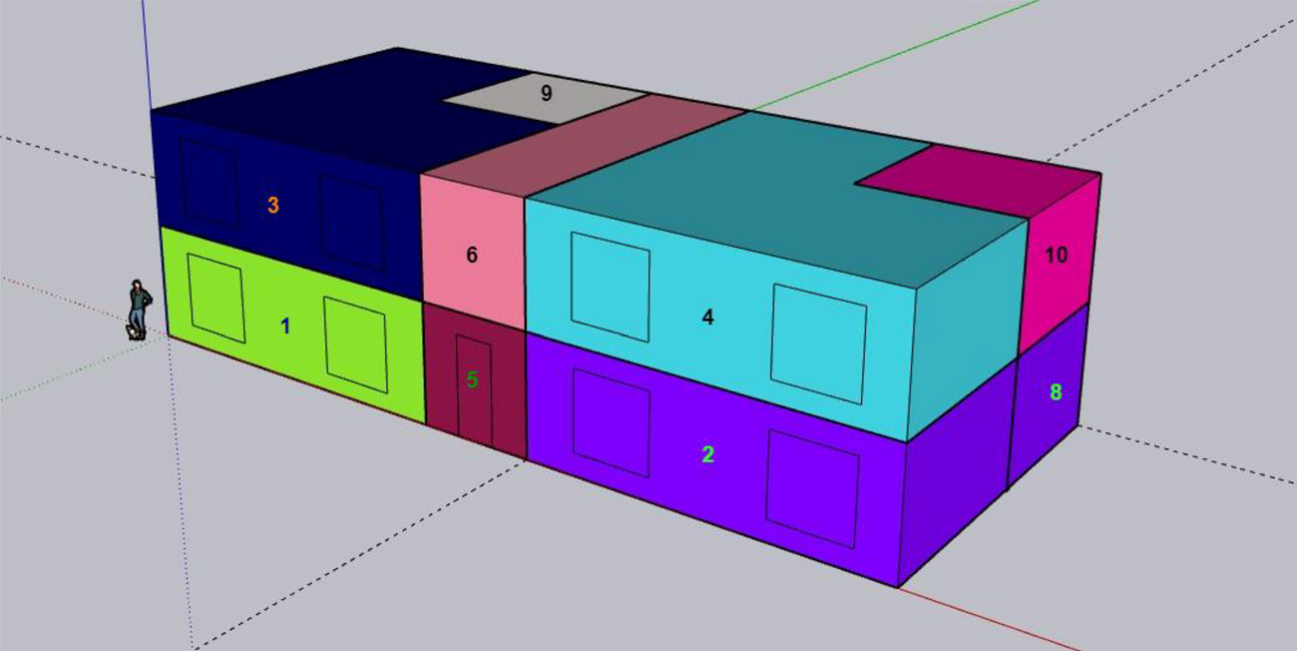
10 Thermal Zones bifurcation in a residential apartment building.

Firstly, the geometry is created through the SketchUp Plug-in. In this phase, an initial IDF file was created containing the initial inputs for the building's geometry and thermodynamics. The objective of this stage is to utilize this drawing tool to define certain dimensions and types of surfaces (walls, doors, windows, ceilings, etc.) between the interior and exterior. Additionally, boundary conditions of surfaces are established. All this collectively is important information regarding thermal performance. Fig. 3 presents the geometry of the 10-zone residential apartment building created in SketchUp for this work. The building considered in this study is a 2-floor traditional block and has a building area of 192.92 $m^{2}$. There are two units per floor, each with two thermal zones, and a corridor between the apartments, as shown in Figs. 3 and 4. The configurations of the building are described in Table 2.

**Table 2:** Building description.

Location		Trois-Rivières, QC Canada (46.3470° N, 72.5785° W)			
Weather		Trois-Rivières's weather file 2021			
Total Area		192.92 $m^{2}$			
Number of Units		4			
Number of Thermal Zones		10			
Unit's Area		82.81 $m^{2}$			
Living Room Area		66.81 $m^{2}$			
Bedroom (Chamber) area		16 $m^{2}$			
Heating System		Electric Baseboard Heater			
Story Height		3 m			

External Walls					

Material	R×mm		Thickness (mm)	R	Conductivity

Stucco 1N	0.0009		25.3	0.02277	1.111
Structural concrete	0.0004		203.3	0.8132	0.25
Expanded Polystyrene	0.035		100	3.5	0.02857
Gypsum Board	0.0065		6.5	0.04225	0.01538

Celling					

Structural concrete	0.004		263	1.052	0.25
Expanded Polystyrene	0.035		101.6	3.556	0.0286

**Fig. 5:**
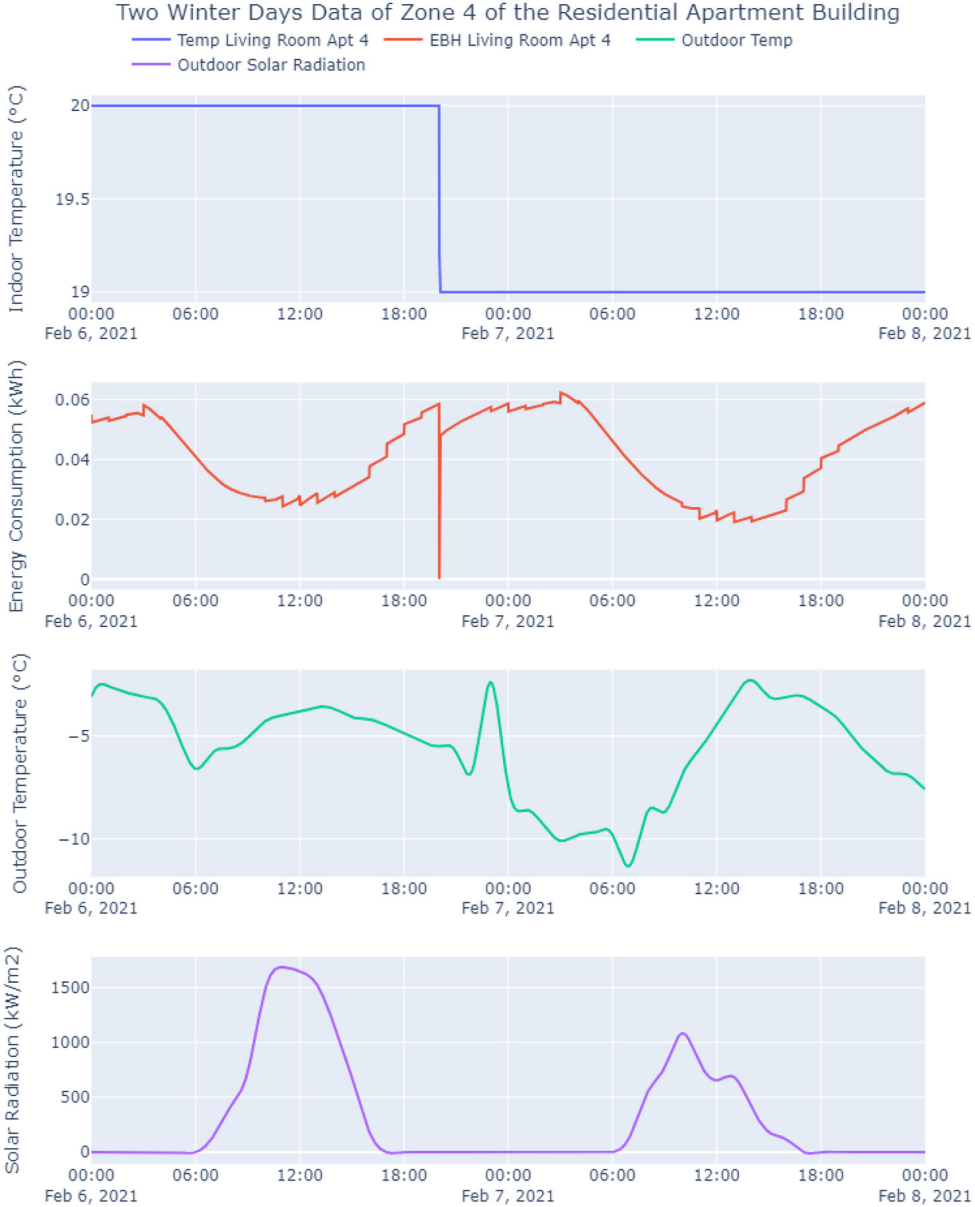
Two winter days data of zone 4 of the residential apartment building.

The building envelope materials satisfy the requirements of ASHRAE climate zone 6 and relevant local standards [11]. After generating .idf from SketchUp, it is imported into OpenStudio to create an OpenStudio model (.osm). Here, the climate data of Trois-Rivieres (.epw) is loaded from the Canadian Weather for Energy Calculations web portal and framed within the ASHRAE handbook fundamentals design regulations. Subsequently, the materials and physical properties are loaded utilizing ASHRAE standards, where the information is validated with documentation from the Canadian Commission on Building and Fire Codes [12]. Electrical loads with different thermal zones are defined to meet the thermal demand. Finally, EnergyPlus is leveraged to conduct the whole-building energy analysis; see Fig. 5 for building behavioural data for two winter days.

### 4.1 Aging correction factors

The aging effect is crucial as the building envelope deteriorates with time, decreasing the thermal insulation and increasing the energy consumption to maintain thermal comfort. Thermal transmittance property is a pivotal property for thermal comfort and energy consumption of thermal zones. In order to address the aging analysis, an estimation of the thermal transmittance $U_{est}\;\left(W/m^{2}K\right)$ is calculated based on weighing factors and reference thermal transmittance $U_{ref}\;\left(W/m^{2}K\right)$ at the time of new construction [13].

The weighing factors are: elapsed time $e_{t}$, finishing material $a_{1}$, color factor $a_{2}$, finishing factor $a_{3}$, distance from sea $d_{s}$, distance from pollution sources $d_{p}$, weather action $w_{a}$. The weighting factors represent the deterioration in outer-wall insulation performance by considering the characteristics identified as influent in this process. The factor $a_{i},i=\left\{1,2,3\right\}$ corresponds to the material quality. Therefore, sub-factor $a_{1}$ is related to the finishing material of the surface. In the construction process, this type of material can be chosen considering the usage of the building. However, components such as bricks, concrete, or stone are highly common in building envelopes. Similarly, sub-factor $a_{2}$ represents the colour of the surface regarding the incidence of dark colours in thermal degradation. Factor $a_{3}$ addresses the tendency of materials with rough surfaces to have higher thermal conductivity rates than materials with smooth surfaces.

The assigned rate for all factors is determined based on the favourability of the conditions as represented in the weighting factors. Unfavourable conditions correlate with an elevated overall thermal transmittance value. The methodology utilized in this work is in accordance with the suggested standard ISO 15686-2 [13], which guides the allocation of values for each weighting factor. These coefficients, assigned to individual subfactors, encapsulate the potential impact on the thermal degradation of the building envelope.

For sub-factor $a_{1}$*,* values are from 0.88 for the most favourable conditions, such as the stone finishing, and 1.1 for bricks, being the most unfavourable scenario; concrete is 1 rated. For sub-factor $a_{2}$*,* two scenarios were considered: the dark colour, being the unfavourable condition, is rated with 1.05, and light colours are qualified as 0.88. For sub-factor $a_{3},$ values represent two kinds of finishing roughness. The favourable scenario is the smooth finishing, which is rated at 0.95. The rough finishing is qualified with a 1.05 as an unfavourable condition.

Factor $d_{s}$*,* the coefficient pertaining to the building's distance from the sea. For cases within a radius of less than 5 km, the rating is 0.88, indicating an increased propensity for thermal deterioration. Conversely, for scenarios beyond this range, the coefficient is 1.1, suggesting a comparatively lower susceptibility to thermal issues. $d_{p}\;$represents the distance to pollution sources, where two types exist: severe and moderate. For the prior, the assigned coefficient is 1.1; for the latter, it is rated at 0.95. Factor $w_{a}\;$is qualified with values ranging from 0.88, representing the most favourable conditions characterized by minimal weather impact, to 1.15, denoting the most unfavourable scenario. Intermediate interactions are assigned a rating of 0.97. The aging calculation methodology [13] can be described by

(1) $$U_{est}=\;\left(e_{t}\cdot a_{1}\cdot a_{2}\cdot a_{3}\cdot d_{s}\cdot d_{p}\cdot w_{a}\right)U_{ref}$$

To estimate the thermal transmittance for aging purposes, the reference thermal transmittance can be modelled as the inverse of thermal resistance based on the National Energy Code of Canada for Buildings standard [11].

(2) $$U_{ref}={\left(R_{thermal}\right)}^{-1}$$

Note that the materials analysed correspond to envelope of the building acting as a barrier between the exterior and interior of the building [14]. The elapsed time $e_{t}$ factor used are: 13-15 years age ($e_{t}=1.28)$, 16-22 years age ($e_{t}=1.39)$, 23-27 years age ($e_{t}=1.54)$, and 28-33 years age ($e_{t}=1.88)$. Moreover, the changes in three factors: distance from sea $d_{s}$, distance from pollution sources $d_{p}$, and weather action $w_{a}$ were introduced to create 3 cases for the residential building in a city. Case 1 represents mild conditions for aging, which implies a lower value for the corresponding weighing factors, indicating that the impact of aging is mitigated. Cases 2 and 3 (moderate and severe) followed the scale of the magnitude described in the methodology of [13], where the external conditions accelerate the building envelope's aging. In all cases, the architectural characteristics of the studied building will remain the same. Thus, Subfactor $a_{i}\;$related to materials, will have the following values: ($a_{1}\;$=1 (Concrete)), ($a_{2}$=1.05 (Light)), and ($a_{3}$=0.95 (smooth)) and the rest are depicted in Table 3.

**Table 3:** Aging cases.

Cases	$d_{s}$	$d_{p}$	$w_{a}$
Mild (Case 1)	0.95	0.95	0.88
Moderate (Case 2)	1.05	1.1	0.97
Severe (Case 3)	1.05	1.1	1.15

**Fig. 6:**
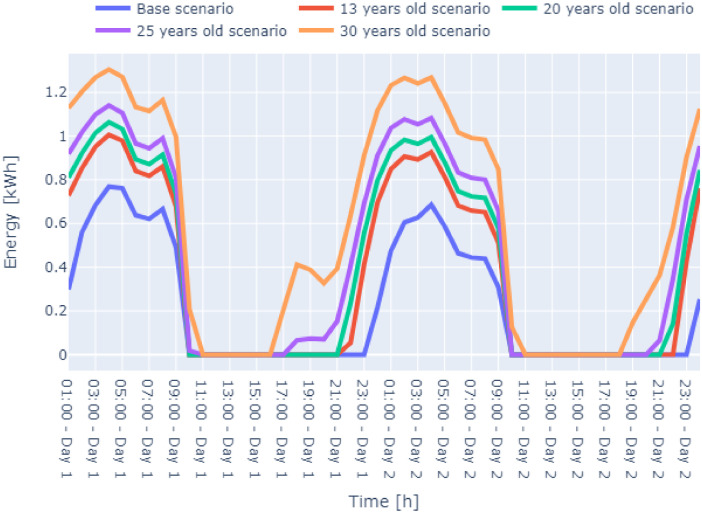
Case 1 – Mild Conditions two-day winter energy consumption data Zone-4 of the residential building.

**Fig. 7:**
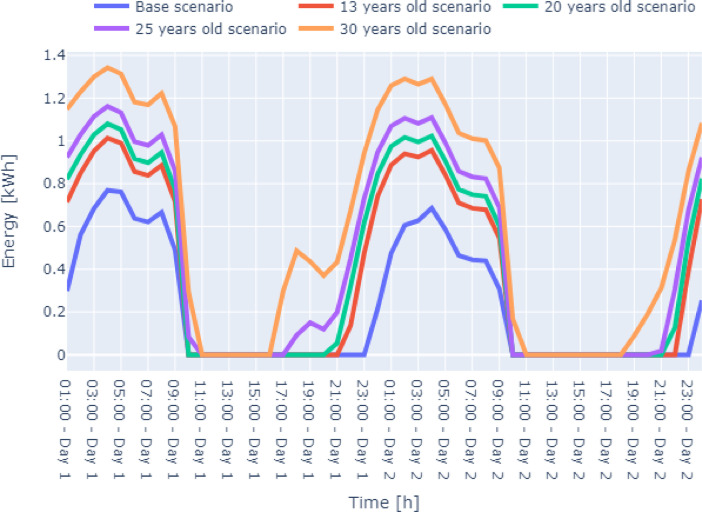
Case 2 – Moderate Conditions two-day winter energy consumption data Zone-4 of the residential building.

**Fig. 8:**
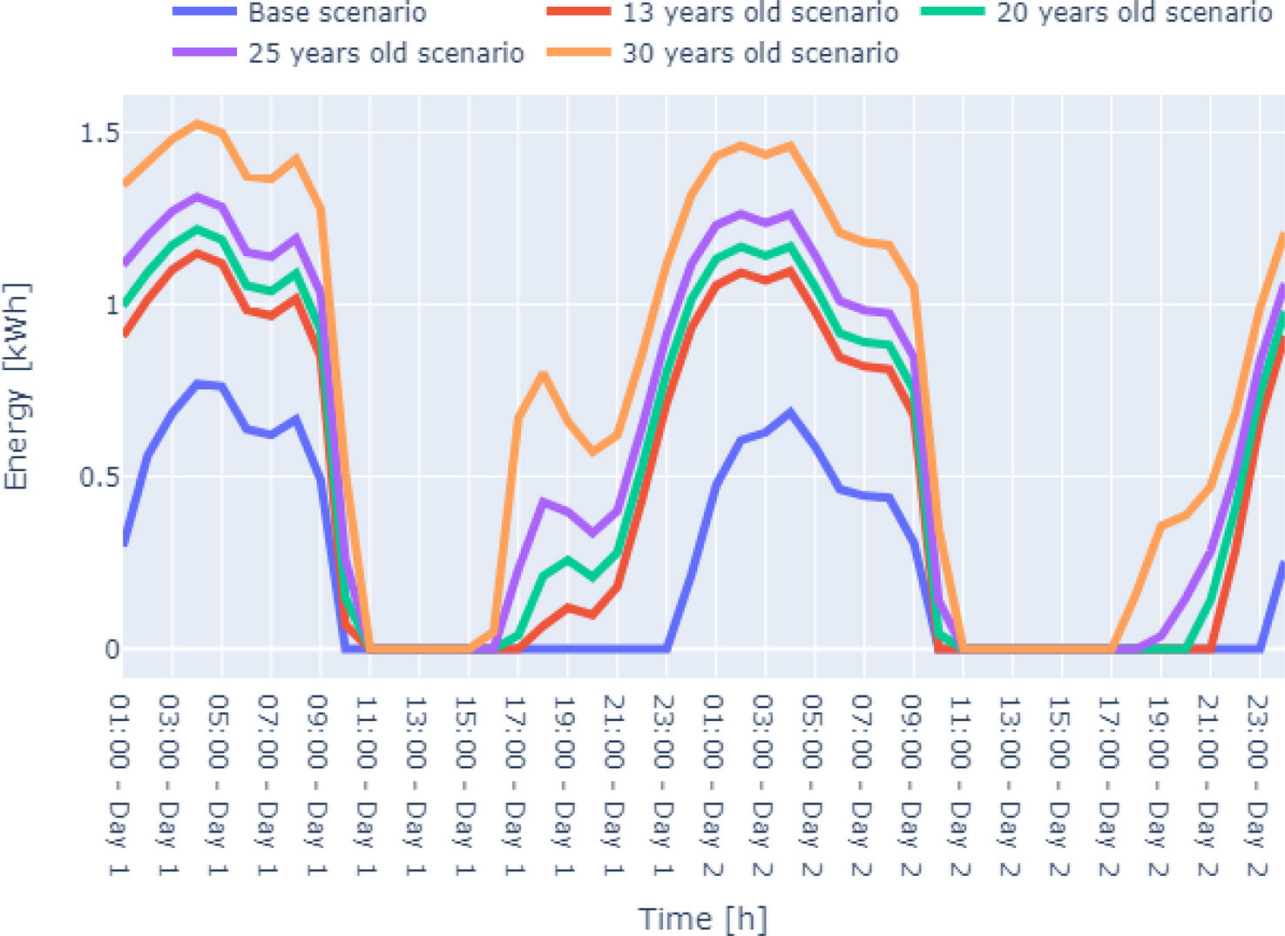
Case 3 – Severe Conditions two-day winter energy consumption data Zone-4 of the residential building.

Figs. 6, Fig. 7, Fig. 8 display the two-winter day energy consumption data of three cases for zone-4 of the residential building.

## Limitations

The construction materials specification and the weather files for the dataset generation belong to Zone 6 of the ASHRAE standard handbook, potentially limiting its applicability to other geographical areas.

## Ethics Statement

The authors confirm that they have read and followed the ethical requirements for publication in Data in Brief and confirm that the current work does not involve human subjects, animal experiments, or any data collected from social media platforms.

## CRediT authorship contribution statement

**Juan Pablo Diaz Ramirez:** Methodology, Data curation, Software, Writing – original draft. **Shaival Hemant Nagarsheth:** Conceptualization, Investigation, Formal analysis, Writing – original draft, Writing – review & editing. **Camilo Enrique Ducuara Ramirez:** Data curation, Visualization. **Nilson Henao:** Resources, Supervision, Writing – review & editing. **Kodjo Agbossou:** Supervision, Project administration, Funding acquisition.

## Data Availability

Energy Consumption and Thermal Modelling Dataset for Multi-Zone Residential Apartment Building (Original data) (Harvard Dataverse).
